# Inhibition of *γ*-secretase induces G2/M arrest and triggers apoptosis in breast cancer cells

**DOI:** 10.1038/sj.bjc.6605034

**Published:** 2009-06-09

**Authors:** S Rasul, R Balasubramanian, A Filipović, M J Slade, E Yagüe, R C Coombes

**Affiliations:** 1 Division of Surgery, Oncology, Reproductive Biology and Anaesthetics, Department of Oncology, MRC Cyclotron Building, Imperial College London, London W12 0NN, UK

**Keywords:** *γ*-secretase, breast cancer, Notch, cell cycle arrest, proteasome, apoptosis

## Abstract

*γ*-Secretase activity is vital for the transmembrane cleavage of Notch receptors and the subsequent migration of their intracellular domains to the nucleus. Notch overexpression has been associated with breast, colon, cervical and prostate cancers. We tested the effect of three different *γ*-secretase inhibitors (GSIs) in breast cancer cells. One inhibitor (GSI1) was lethal to breast cancer cell lines at concentrations of 2 *μ*M and above but had a minimal effect on the non-malignant breast lines. GSI1 was also cytotoxic for a wide variety of cancer cell lines in the NCI60 cell screen. GSI1 treatment resulted in a marked decrease in *γ*-secretase activity and downregulation of the Notch signalling pathway with no effects on expression of the *γ*-secretase components or ligands. Flow cytometric and western blot analyses indicated that GSI1 induces a G2/M arrest leading to apoptosis, through downregulation of Bcl-2, Bax and Bcl-XL. GSI1 also inhibited proteasome activity. Thus, the *γ*-secretase inhibitor GSI1 has a complex mode of action to inhibit breast cancer cell survival and may represent a novel therapy in breast cancer.

*γ*-Secretase is an aspartyl protease macromolecular complex comprising nicastrin (NCSTN), anterior pharynx-defective 1-a/b/c isoforms, the enzymic component presenilin-1/2 (PSEN) and presenilin enhancer protein-2 (PSENEN, Pen-2). By providing an internally hydrophilic environment within the plasma membrane (Lazarov *et al*, 2006), *γ*-secretase is responsible for cleaving more than 30 substrates, including amyloid precursor protein (APP), Notch (Mumm *et al*, 2000), ErbB4 (Ni *et al*, 2001), E-cadherin (Marambaud *et al*, 2002), CD44 (Lammich *et al*, 2002) and p75 (Schluesener *et al*, 1992), which regulate vital cell functions such as proliferation, cell cycle, cell adhesion and apoptosis. Interestingly, increased expression of Notch ligands, receptors, and/or downstream targets are highly associated in the pathogenesis of breast (Jones *et al*, 2004; Stylianou *et al*, 2006; Yamaguchi *et al*, 2008), brain (Purow *et al*, 2005), colon (Akiyoshi *et al*, 2008), cervical (Liu *et al*, 2007) pancreatic (Doucas *et al*, 2008) and skin cancers (Dang *et al*, 2006). Thus, the *γ*-secretase complex may be a potential therapeutic target in a wide array of carcinomas.

Notch signalling is initiated through the interactions between the plasma-embedded Notch heterodimer receptors and cell surface ligands (Jagged-1, -2, Delta-like -1, -2 and -4) present on adjacent cells (Lindsell *et al*, 1995). This results in a conformational change in Notch to reveal the site 2 cleavage site for metalloproteases (ADAM10, ADAM17), which leaves a 12 amino-acid stub of the Notch extracellular domain, required for subsequent recognition and cleavage by the *γ*-secretase complex (Brou *et al*, 2000; Mumm *et al*, 2000). *γ*-Secretase cleavage of Notch liberates the intracellular domain (NICD), which translocates to the nucleus where it binds to the CSL family of DNA-binding proteins (CBF1/RBPJ-*κ* in mammalian cells) (Tamura *et al*, 1995). The NCID–CBF1 complex initiates recruitment of transcriptional co-activators Mastermind-like-1, 2, 3, and p300 (Oswald *et al*, 2001) to enable gene transcription of Notch downstream targets. These include *Hes*, *Hey* (Iso *et al*, 2003) *c-myc* (Klinakis *et al*, 2006), *p21* (Rangarajan *et al*, 2004) and *cyclin D1* (Ronchini and Capobianco, 2001), which regulate proliferation, differentiation and apoptosis.

Notch activation has been observed in a number of malignancies. It was first reported when insertion of mouse mammary tumour virus within the Notch 4 locus incited tumour formation in mice (Gallahan *et al*, 1996). In humans, elevated Notch1 is observed in Ras-positive breast cancers (Weijzen *et al*, 2002), while both increased Notch1 and Jagged1 expression decreases patient survival (Jones *et al*, 2004; Reedijk *et al*, 2005; Dickson *et al*, 2007). In addition, Numb, a negative regulator of Notch signalling (McGill and McGlade, 2003), is significantly reduced in 50% of breast cancer tissues, inversely correlating with tumour size (Pece *et al*, 2004) and correlating with a poor prognosis (Colaluca *et al*, 2008). Inhibiting Notch signalling by overexpression of Numb reverts the transformed phenotype of MCF-7 cells (Stylianou *et al*, 2006), whereas silencing of Notch 3 inhibits proliferation and promotes apoptosis in ErbB2-negative breast cancer cell lines (Yamaguchi *et al*, 2008). Moreover, overexpression of Notch in non-tumourigenic breast epithelial cells induces transformation *in vitro* (Imatani and Callahan, 2000; Stylianou *et al*, 2006). Thus, deregulation of Notch signalling plays a significant role in tumorigenesis.

Synthetic γ-secretase inhibitors have been successful in treating Alzheimer's disease, where defective *γ*-secretase cleavage of the substrate molecule APP results in a longer A*β*42 variant of A*β*40 peptides, consequently leading to plaque formation (Lichtenthaler *et al*, 1997). In breast cancer, the *γ*-secretase inhibitor DAPT (N-[N-(3,5-Difluorophenacetyl-L-alanyl)]-S-phenylglycine *t*-butyl ester) has been shown to effectively reduce DCIS-induced mammosphere formation (Farnie *et al*, 2007). We therefore investigated the effects of commercially available *γ*-secretase inhibitors on the proliferation/survival of normal and malignant breast cancer cell lines, their effects on *γ*-secretase component expression and the possible mechanisms involved. We show here that inhibition of *γ*-secretase activity in breast cancer cell lines induces G2/M arrest and downregulation of antiapoptotic proteins leading to cell death.

## Materials and methods

### Cell culture

Oestrogen receptor-*α* (ER)-positive (MCF-7, T47D, and ZR-75-1) and ER-negative (MDA-MB-231 and CAL-51) breast cancer cell lines were cultured in Dulbecco's modified Eagle's medium supplemented with 10% foetal calf serum (FCS) and 2 mM L-glutamine, 100 U ml^−1^ penicillin, 0.1 mg ml^−1^ streptomycin. Non-tumorigenic 226-L-U19 and 226-L-TS4 breast cell lines were kindly provided by Parmjit S Jat, Ludwig Institute for Cancer Research, London, and were cultured in DME/F12 medium supplemented with 10% FCS containing, 5 *μ*g ml^−1^ insulin, 1 *μ*g ml^−1^ hydrocortisone, 10 ng ml^−1^ epidermal growth factor, 10 ng ml^−1^ cholera toxin and 2 mM L-glutamine, 100 U ml^−1^ penicillin, 0.1 mg ml^−1^ streptomycin.

### Effect of *γ*-secretase inhibitors on proliferation assays

The *γ*-secretase inhibitors, DAPT, Compound E ((2S)-2-{[(3,5-Difluorophenyl)acetyl]amino}-*N*-[(3S)-1-methyl-2-oxo-5-phenyl-2,3-dihydro-1*H*-1,4-benzodiazepin-3-yl]propanamide) and *γ*-secretase inhibitor 1 (GSI1: z-Leu-Leu-Nle-CHO), were purchased from Merck Biosciences (Darmstadt, Germany). Cells (2.5 × 10^3^) were seeded in triplicate and allowed to adhere overnight. The media was replaced with media containing the inhibitors diluted in dimethyl sulphoxide (DMSO). After 48 h, the media was removed and the cells were harvested and counted using a Coulter Counter (Beckman Coulter, Buckinghamshire, UK).

### Sulphorhodamine B assay

The sulphorhodamine B (SRB) assay was used to screen for GSI1 cytotoxicity (Vichai and Kirtikara, 2006). The assay relies on the ability of SRB to bind to protein components of cells that have been fixed to tissue-culture plates by trichloroacetic acid (TCA). SRB is a bright-pink aminoxanthene dye with two sulphonic groups that bind to basic amino-acid residues under mild acidic conditions, and dissociate under basic conditions. As the binding of SRB is stoichiometric, the amount of dye extracted from stained cells is directly proportional to the cell mass. To determine the effect of GSI1 on cell number over time, SRB assays were performed as described. Cells were seeded with six replicates in flat-bottomed 96-well plates (3000 cells per well). The cells were allowed to adhere overnight, and then media containing GSI1 at different concentrations were added. One plate was assayed at this time point (time zero) and further plates were assayed at 2-day intervals until day 10. The cells were fixed by adding 100 *μ*l per well of ice-cold 40% TCA to each well for 60 min. The plates were washed five times in running tap water and stained with 100 *μ*l per well SRB reagent (0.4% w/v SRB (Sigma-Aldrich, Poole, UK)) in 1% acetic acid for 30 min. The plates were washed five times in 1% acetic acid and allowed to dry overnight. SRB was solubilised with 100 *μ*l per well 10 mM Tris-base, shaken for 30 min and the optical density measured at 492 nm.

### *γ*-Secretase activity assay

Cells (1.5 × 10^6^) were treated with increasing concentrations of GSI1 (0.75, 2, and 5 *μ*M) and *γ*-secretase activity was measured after 24 and 48 h using the R&D Systems (R&D, Minneapolis, MN, USA) *γ*-Secretase Activity Kit following the manufacturer's instructions. Briefly, cells were washed twice with ice-cold phosphate-buffered saline (PBS), harvested in the cell extraction buffer, and incubated on ice for at least 10 min. Lysates were centrifuged at 10 000 **g** for 1 min and supernatants were collected with a total protein yield of 0.5–1.0 mg ml^−1^. Pierce BCA Protein Assay (Pierce, Rockford, IL, USA) was used to determine the protein concentration in each sample. Protein (200 *μ*g) was incubated with the *γ*-secretase fluorogenic substrate for 2 h at 37°C and fluorescence was measured at 355/460 nm.

### CBF-1 reporter assay

The ability of the *γ*-secretase-cleaved NICD to bind to CBF1 and activate gene transcription was measured by the transfection of a reporter in which four copies of the wild-type CBF1-binding elements were cloned in front of a simian virus 40 promoter-driven luciferase gene (4xwtCBF1Luc) (Hsieh *et al*, 1996). Twenty-four hours after transfection, cells were treated with GSI1 and luciferase activity was measured 24 h later using a Promega Luciferase kit (Promega, Madison, WI, USA) following the manufacturer's recommendations.

### National Cancer Institute Screen of *γ*-secretase inhibitor

The human tumour cell lines of the cancer-screening panel (NCI60) were cultured in RPMI 1640 medium containing 5% foetal bovine serum and 2 mM L-glutamine. Cells were inoculated into 96-well microtiter plates in 100 *μ*l at plating densities ranging from 5000 to 40 000 cells per well, depending on the doubling time of individual cell lines. After cell inoculation, the microtiter plates were incubated at 37°C, 5% CO_2_ for 24 h before addition of experimental drugs. After 24 h, two plates of each cell line were fixed *in situ* with TCA to represent a measurement of the cell population for each cell line at the time of drug addition (Tz). GSI1 in complete medium containing 50 *μ*g ml^−1^ gentamicin was added to the cell lines and the plates were incubated for an additional 48 h at 37°C, 5% CO_2_, and an SRB assay was performed. Using the seven absorbance measurements (time zero (Tz), control growth (C), and test growth in the presence of drug at the five concentration levels (Ti)), the percentage growth was calculated at each of the drug concentrations levels. Percentage growth inhibition was calculated as: 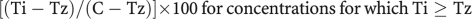




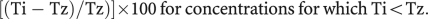



Three dose–response parameters were calculated for each experimental agent. Growth inhibition of 50% (GI50) was calculated as was the drug concentration resulting in total growth inhibition (TGI) is calculated from Ti=Tz. The LC_50_ (concentration of drug resulting in a 50% reduction in the measured protein at the end of the drug treatment as compared with that at the beginning) indicating a net loss of cells following treatment is calculated from [(Ti−Tz)/Tz] × 100=−50. Values were calculated for each of these three parameters if the level of activity is reached; however, if the effect is not reached or is exceeded, the value for that parameter is expressed as greater or less than the maximum or minimum concentration tested.

### Cell cycle analysis

Cells (3 × 10^5^) were plated out and after 24 h the media was changed to one containing DMSO, or GSI1 at a concentration of 0.75, 1, or 5 *μ*M. Both adherent and floating cells were harvested after 24 and 48 h, fixed with ice-cold 70% methanol, washed twice with 5 ml PBS, treated with 10 *μ*g RNAse, and stained with 50 *μ*g propidium iodide, and their DNA content was estimated by flow cytometry using a Beckton Dickinson FacsCanto flow cytometer (Beckton Dickinson, Franklin Lakes, NJ, USA) to determine the proportion of cells at each stage of the cell cycle.

### RNA expression

Cells were lysed with 4 M guanidinium isothiocyanate and total RNA was extracted using RNAeasy (Qiagen Ltd, West Sussex, UK) according to the manufacturer's instructions. cDNA was synthesised from 2 *μ*g of RNA with Moloney Murine Leukemia Virus reverse transcriptase and amplified by PCR on a LightCycler (Roche Diagnostics, Mannheim, Germany) using the LightCycler DNA FastStart SYBR Green 1 kit (Roche Diagnostics). Oligonucleotide sequences and PCR conditions are described in detail in Supplementary Table 1.

### Protein expression

Cell lines were harvested and lysed in Lysis buffer (150 mM NaCl, 0.1% SDS, 5 mM EDTA, 10 mM Tris (pH 7.2), 1% Triton-X, and 1% deoxycholate), containing 1 mM phenylmethanesulphonylfluoride and protease inhibitor cocktail (Sigma-Aldrich P8340: AEBSF 104 mM, Aprotinin 0.08 mM, Leupeptin 2 mM, Bestatin 4 mM, Pepstatin A 1.5 mM, E-64 1.4 mM). Whole cell extracts were denatured at 100°C for 10 min and were electrophoresed on a 10% SDS–polyacrylamide gel. Primary antibodies Bcl-XL, Bcl-2, Bax and XIAP (Beckton Dickinson), Notch 1 intracellular domain (Abcam Plc, Cambridge, UK), and *γ*-tubulin (Sigma-Adrich) were incubated in 0.1% TBS-Tween overnight. Blots were visualised using chemiluminescence (ECL) (Amersham Bioscience, Buckinghamshire, UK), according to the manufacturer's instructions.

### Proteasome activity

The inhibitory effect of GSI1 and MG132 (z-Leu-Leu-Leu-CHO) on the 20S proteasomal component (Millipore, Billerica, MA, USA) was determined *in vitro* after pre-incubation for 15 min at room temperature. Then, the enzyme-inhibitor mix was added to 1 × assay buffer (25 mM HEPES, 4-(2-hydroxyethyl)-1-piperazineethanesulphonic acid, pH 7.5, 0.5 mM EDTA, 0.05% (v/v) NP-40 and 0.001% (w/v) SDS), and incubated with N-Succinyl-Leu-Leu-Val-Tyr-7-Amino-4-methylcoumarin (suc-LLVY-AMC, Millipore) for 75 min at 40°C. Three replicates were included per treatment. Fluoresence was measured at 380/460 nm.

The effect of GSI1 and MG132 on proteasomal activity *in vivo* was determined after treatment of MDA-MB-231 and MCF-7 cells (1 × 10^6^) with the inhibitors for 4 h. Adherent cells were washed and scraped in cold PBS, collected and centrifuged for 5 min at 170 × **g** and 4°C. Cells were resuspended in 50 mM HEPES, pH 7.5, 5 mM EDTA, 150 mM NaCl and 1% Triton X-100, and incubated on ice for 30 min, with vortexing at 10 min intervals. Samples were centrifuged at 14 000 **g** for 15 min at 4°C, and the supernatant was collected. Lysates were incubated with 1 × assay buffer and 50 *μ*M suc-LLVY-AMC at 40°C for 75 min. Three replicates were included per treatment. Fluorescence was measured at 380/460 nm.

### Statistical analysis

Where indicated a *t*-test was performed (two-sided). Statistical significance was assumed when *P*<0.05.

## Results

### Downregulation of the Notch signalling pathway with GSI1 selectively affects the viability of breast cancer cells

**Figure 1:**
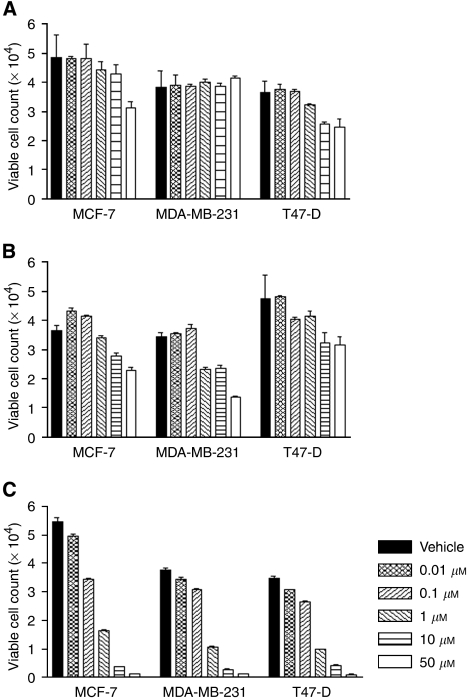
Cytotoxic effect of *γ*-secretase inhibitors on breast cancer cell lines. Cells were treated with Compound E (**A**), DAPT (**B**), and GSI1 (**C**) for 48 h and their viability was determined using a Coulter counter. Data represent the average of three independent experiments ±s.d.

**Figure 2:**
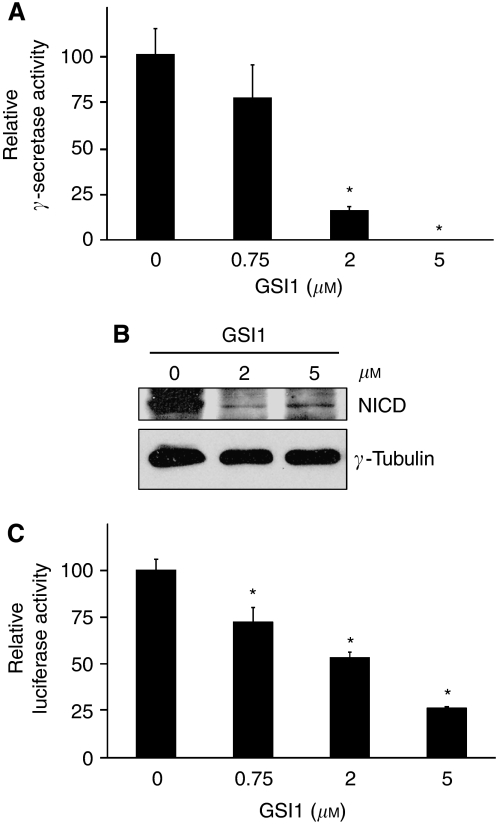
GSI1 downregulates *γ*-secretase activity and the Notch pathway. (**A**) Relative *γ*-secretase activity in MCF-7 cells were treated with GSI1 for 24 h. (**B**) Western blot analysis of NICD in MCF-7 cells were treated with GSI1 for 24 h. (**C**) MCF-7 cells were transfected with a *CBF1*-luciferase reporter and the next day treated with GSI1 for 24 h. Values represent relative luciferase activity with respect to the mock-transfected and vehicle-treated cells. Bars indicate the average *γ*-secretase (**A**) or luciferase activity (**C**) ±s.d. of three independent experiments (^*^ indicates *P*<0.05).

The breast cancer cell lines MDA-MB-231, T47D, and MCF-7 were treated with three *γ*-secretase inhibitors at concentrations in the range 0.01–50 *μ*M. Compound E had no effect on the MDA-MB-231 cells and only reduced the proliferation of the other cell lines by less than 50% at a concentration of 50 *μ*M (Figure 1A). The effect of DAPT on T47D and MCF-7 cell lines was comparable with the Compound E, but DAPT inhibited proliferation by approximately 50% in the MDA-MB-231 lines at 50 *μ*M (Figure 1B). GSI1 had the most significant effect on all three cell lines at 1 *μ*M reducing proliferation by approximately 80% (Figure 1C). GSI1 inhibited *γ*-secretase activity in MCF-7 cells in a dose-dependent manner and at 5 *μ*M inhibition reached 100% (Figure 2A).

We confirmed that inhibition of *γ*-secretase by GSI1 downregulated the Notch pathway by directly detecting the NICD (which is cleaved by *γ*-secretase) with a specific antibody. Treatment of MCF-7 cells with 2 and 5 *μ*M GSI1 decreased notably the levels of NICD (Figure 2B). It is well established that the Notch intracellular domain interacts with the transcriptional repressor CBF1 and abolishes CBF1-mediated repression. We used a surrogate for the activation of Notch by *γ*-secretase consisting of the transfection of a luciferase reporter containing CBF1-binding sites (Hsieh *et al*, 1996). In this system, activation of Notch is reflected by an increase in luciferase expression. Treatment of MCF-7 cells with GSI1 reduced the luciferase activity of the transfected reporter in a dose-dependent manner (Figure 2C). Interestingly, the effect of GSI1 on Notch-driven luciferase transcription was always lower than the corresponding inhibition of *γ*-secretase activity, probably due to a less than 100% transfection efficiency and activation of *CBF1-*Luciferase by Notch-independent mechanisms.

**Figure 3:**
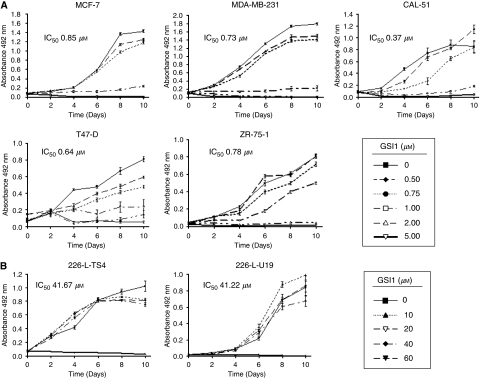
Cytotoxic effect of GSI1 on breast cancer cells. The effect of increasing concentrations of GSI1 in breast cancer cell lines (**A**) and non-tumorigenic breast cell lines (**B**) was determined by staining with SRB. Data represents the average ±s.d. of two independent experiments with six replicates at each time point. The IC_50_ for each cell line was determined from the corresponding dose–response curves (data not shown) and is indicated.

Sulphorhodamine B assays were then carried out on five tumorigenic and two non-tumorigenic breast cell lines to investigate the effects of GSI1 over a longer time frame. In MCF-7, MDA-MB-231, ZR-75-1, T47D and CAL-51 breast cancer cell lines 1 *μ*M GSI1 and above resulted in cell death (range of IC_50_ values: 0.6–0.9 *μ*M) (Figure 3A). No effect on the non-tumorigenic 226-L-U19 and 226-L-TS4 cell lines was seen in the range 0.5–40 *μ*M, which showed IC_50_ values around 50 *μ*M (Figure 3B).

There was no effect on the expression of the *γ*-secretase components or ligands such as Notch, Jagged, and Numb in response to the GSI1 treatment (Supplementary Figure 1). Thus, the effect of GSI1 on breast cancer cells is not due to a differential expression of *γ*-secretase components or its downstream effectors.

### Effect of GSI1 on the NCI 60 panel of cancer cell lines

**Figure 4:**
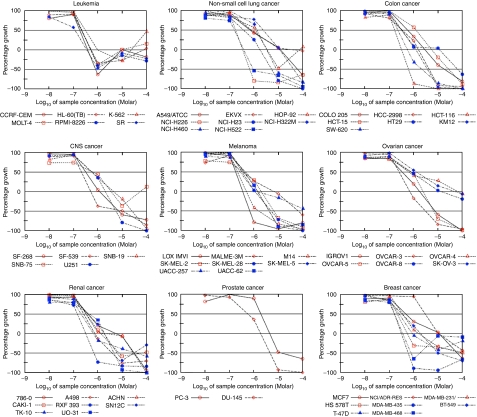
Cytotoxic effect of GSI1 on the NCI60 panel of cancer cell lines. The cytotoxic effect of GSI1 (10, 100 nM, 1, 10, 100 *μ*M) on cancer cells was determined by a SRB assay after 24 h of drug treatment. Although NCI/ADR-RES data are shown within the breast cancer cell panel, recent evidence suggests that this is an ovarian cancer multidrug-resistant line derived from OVCAR-8 (Liscovitch and Ravid, 2007). Results shown are representative of two independent experiments.

The effect of GSI1 (10 nm, 100 nM, 1, 10, and 100 *μ*M) was tested, in duplicate, on the NCI panel of 60 cancer cell lines. A decrease in cell number was seen in all 60 cell lines with the mean log_10_ GI_50_ of −6.13±0.007 M (Figure 4). The mean log_10_ LC_50_ calculated for all of the cell lines was −4.41±0.011 M (Supplementary Table 2). When the screen was repeated the values obtained were −6.41±0.005 M for the log_10_ GI_50_, and −4.49±0.12 M for the log_10_ LC_50_.

When the breast cancer cell lines only were examined the mean log_10_ GI_50_ were −6.23±0.063 and −6.58±0.059 M (on the second run the MDA-MB-468 cell line was included). The mean values of log_10_ LC_50_ were −5.12±0.078 and −4.86±0.12 M in runs 1 and 2, respectively. GSI1 had a negligible effect on cell number at 100 nM, but at concentration of 1 *μ*M and higher it had an inhibitory effect (Figure 4). Interestingly, the multidrug-resistant cell line NCI/ADR-RES, resistant to adriamycin and other P-glycoprotein substrates, was less sensitive to GSI1 than the other breast cancer cell lines tested and than most other cancer cell lines. At 1 *μ*M, GSI1 did not show any decrease in cell number, which was only noticeable at 10 *μ*M (Figure 4). Thus, GSI1 inhibits the proliferation of many different cancer cell lines corresponding to a variety of solid tumours and leukaemia.

### GSI1 induces a G2/M arrest resulting in apoptosis

**Figure 5:**
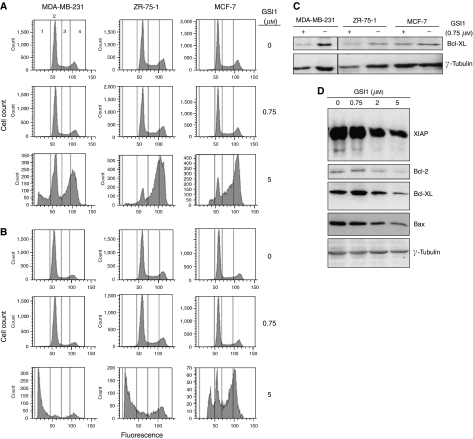
GSI1 induces G2/M cell cycle arrest and triggers apoptosis in breast cancer cells. (**A**, **B**) Cell cycle analyses of breast cancer cell lines treated with GSI1 for 24 h (**A**) and 48 h (**B**). Cells were stained with propidium iodide and gated according to their fluorescence to differentiate cell cycle phases: 1, apoptotic cells; 2, cells in G0/G1; 3, cells in S phase; 4, cells in G2-M. Data shown are a representative of two independent experiments. (**C, D**) Western blot analyses were used to verify the modulation of the apoptotic response due to GSI1 treatment. (**C**) Three different breast cancer cell lines treated with a single GSI1 concentration (0.75 *μ*M) for 48 h. (**D**) MCF-7 cells treated for 48 h with increasing concentrations of GSI1. Tubulin was used as a loading control. Note the slight effect of GSI1 on tubulin levels in MDA-MB-231 and ZR-75-1 cells (**C**).

We observed a marked increase in the percentage of cells in G2/M arrest after 24 h incubation with 5 *μ*M GSI1 (Figure 5A). After 48 h treatment, a large proportion of the ZR-75-1 and MDA-MB-231 cells were apoptotic (58 and 77%, respectively) and only a small percentage were in G2/M arrest (Figure 5B). Seventeen percent of MCF-7 cells were apoptotic after treatment with 5 *μ*M GSI1 (Figure 5B), but there was a substantial increase in the percentage of cells in G2/M arrest. Media replacement experiments indicated that when fresh medium was added to MDA-MB-231 cells previously treated with GSI1 for 48 h, the cells recovered their proliferative capacity when concentration was up to 1 *μ*M. However, at higher concentrations no further proliferation was observed (data not shown). When the levels of the antiapoptotic protein Bcl-XL were monitored in MDA-MB-231, ZR-75-1, and MCF-7 cells, we found a downregulation due to GSI1 treatment (0.75 *μ*M for 48 h) in the two former cell lines (Figure 5C). However, GSI1 treatment in MDA-MB-231 and ZR-75-1 cell lines also downregulated the level of *γ*-tubulin (Figure 5C) as well as other proteins tested as loading controls (eIF4E, RPLP0, ribosomal protein S6, among others). We then monitored the levels of the antiapoptotic proteins XIAP, Bcl-2, Bax and Bad after treatment with increasing doses of GSI1 and found that their levels decreased progressively from 2 to 5 *μ*M GSI1 (Figure 5D). Thus, GSI1 triggers apoptosis in breast cancer cells by downregulating the expression of antiapoptotic proteins.

### GSI1 inhibits proteasome activity both *in vivo* and *in vitro* but has less cytotoxic effect on breast cancer cells than MG132

**Figure 6:**
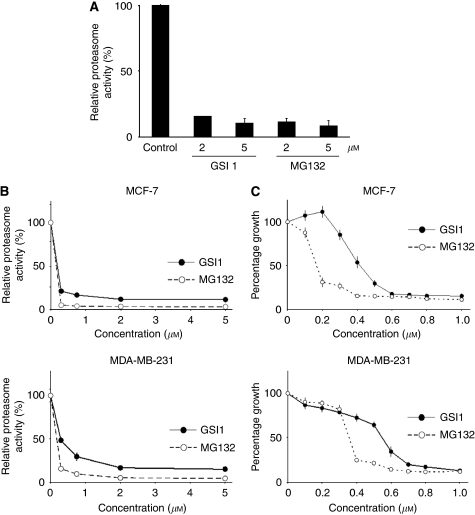
GSI1 inhibits proteasome activity both *in vivo* and *in vitro* but has less cytotoxic effect on breast cancer cells than MG132. (**A**) Inhibition of proteasome activity *in vitro*. The 20S proteasomal component was pre-incubated with GSI1, MG132, or vehicle for 15 min at room temperature, and then the proteolysis of suc-LLVY-AMC was monitored by fluorimetry. One unit of activity was defined as the amount of protein producing one *μ*mol of AMC per minute at 40°C. Data represent the average ±s.d. of two experiments. (**B**) Effect of both GSI1 and MG132 on proteasome activity in cell extracts from breast cancer cells. Both drugs where added to the culture medium of breast cancer cells, and after 4 h, proteasome activity was determined in cell extracts by proteolysis of suc-LLVY-AMC. Data represent the average ±s.d. of three experiments. (**C**) Cytotoxic effect of GSI1 and MG132 on breast cancer cells. The effect of increasing drug concentrations on breast cancer cells was determined by staining with SRB after 48 h. Data represent the average ±s.d. of two independent experiments with six replicates at each time point.

As GSI1 (z-Leu-Leu-Nle-CHO) is chemically and structurally similar to proteasomal inhibitor MG132 (z-Leu-Leu-Leu-CHO), the possibility of GSI1 affecting proteasomal activity was explored. Proteasomal activity was severely reduced (80-90%) upon incubation treatment of the isolated proteasome 20S proteolytic core particle subunit with either compound *in vitro* (Figure 6A). Similarly, a strong inhibitory effect was observed *in vivo* (Figure 6B), although MG132 was marginally more efficient than GSI1. However, the cytotoxic effect of both compounds on both MCF-7 and MDA-MB-231 cells was markedly different (Figure 6C), MG132 showing a stronger cytotoxic effect than GSI1 (between 1.5- and 2-fold). Thus, despite their similar chemical structure and protesome inhibition, GSI1 and MG132 affect the growth of breast cancer cells differently.

## Discussion

In this study we endeavoured to determine whether the *γ*-secretase complex, which has an integral role in signalling of Notch, is a potential therapeutic target in breast cancer. For this we tested the effect of commercial *γ*-secretase inhibitors on breast cell lines. The three *γ*-secretase inhibitors tested had markedly different effects on different breast cancer cell lines. Both DAPT and Compound E are chemical inhibitors of the complex. DAPT reduces ductal carcinoma *in situ* mammosphere formation (Farnie *et al*, 2007) and pancreatic cancer cell growth (Kimura *et al*, 2007). Another *γ*-secretase inhibitor, MRK-003, reduces tumour cell proliferation, inhibits serum independence, and induces apoptosis of lung cancer cell lines *in vitro* and *in vivo* using mouse xenograft models (Konishi *et al*, 2007). Thus, the *γ*-secretase complex is now becoming an accepted target in cancer therapy, in particular, with regard to Notch signalling (Shih and Wang, 2007).

Differential responses between tumourigenic and non-tumourigenic cell lines may be explained by differential expression of Numb, a negative regulator of the Notch pathway, and NICD. It has been shown that non-tumourigenic cells express Numb but not NICD (Stylianou *et al*, 2006) indicating that, as expected, the Notch pathway is not activated in non-cancerous cells. Conversely, cancer cells have Numb downregulated, NICD upregulated and the Notch pathway activated (Stylianou *et al*, 2006), and are sensitive to the cytotoxic effect of GSI1 by its effect on the Notch pathway.

We show here that *γ*-secretase inhibition promotes a cell cycle arrest at G2/M, which further triggers the apoptotic response. Expression of cyclin B1, which controls the G2/M checkpoint, can be regulated by the Notch pathway (through putative CBF-1-binding elements in its promoter). Breast cancer cells in which the Notch pathway has been targeted, either by an inhibitor of *γ*-secretase or by Notch-1 RNAi, downregulate cyclin B1 and suffer G2/M arrest (Rizzo *et al*, 2008). In addition, in MCF-7 cells another *γ*-secretase inhibitor triggers the DNA damage response with the concomitant upregulation of the cell cycle regulators, p53 and p21, which may promote defective cell division, consequently abrogating antiapoptotic mechanisms (Alimirah *et al*, 2007). We observed a dose-dependent downregulation of Bcl-2, Bax, Bad and XIAP upon GSI1 treatment (Figure 5) and a corresponding dose-dependent activation of caspase 3/7 in MDA-MB-231 cells (data not shown). Increased apoptosis upon treatment with a *γ*-secretase inhibitor has also been observed in Kaposi sarcoma, multiple myeloma (Nefedova *et al*, 2008), melanoma (Leggas *et al*, 2004) and tongue carcinoma (Yao *et al*, 2007). This may be indicative of a possible mechanism through which inhibition of *γ*-secretase modulates decreased viability, as observed in the comprehensive NCI screen.

Notch and APP are probably the best-studied *γ*-secretase substrates, and we have shown that GSI1 treatment downregulates the Notch pathway in breast cancer cells. However, as *γ*-secretase acts upon a large variety of substrates, it is likely that the cytotoxic effect of GSI1 upon cells will be due to the downregulation of several downstream targets involved in vital cell functions, which ultimately affect the survival of cancer cells.

Interestingly, NCI/ADR-RES, multidrug-resistant cells overexpressing the ABC transporter ABCB1 (P-glycoprotein) (Raguz *et al*, 2008) and prostate PC-3 cells, are the least susceptible of all the cells in the NCI-60 cell panel. As PC-3 cells express also P-glycoprotein (Rao *et al*, 2005), and considering the molecular structure of GSI1, it is possible that GSI1 will be a substrate of P-glycoprotein (Crivori *et al*, 2006). This would make GSI1 less effective in cancers from tissues expressing P-glycoprotein such as those from colon or the adrenal gland, or in those which have acquired P-glycoprotein-mediated drug resistance (Burger *et al*, 2003).

We also showed that GSI1 is a potent proteasomal inhibitor, and may inhibit breast cancer cell proliferation through dual targets of the *γ*-secretase complex and the proteasome. Whether GSI1 targets each specifically, or inhibition occurs through sequential consequence remains uncertain. It is tempting to speculate that aldehyde-based compounds, such as GSI1 and MG132, may be able to bind interchangeably between the two complexes. Although a low-resolution electron microscopy model of the *γ*-secretase complex has recently been described (Lazarov *et al*, 2006), only the complete high-resolution X-ray structure of the *γ*-secretase complex may suggest putative shared 3D regions to which aldehyde-based inhibitors bind. In addition, it is possible that cross-talk between the *γ*-secretase complex and the proteasome exists, as PSEN may be cleaved into the active C- and N-terminal fragments by the proteasome (Massey *et al*, 2005). Furthermore, it has been suggested that the *γ*-secretase complex can act in a similar way to the proteasome, as they can recognise, capture substrates and feed them through the proteolytic-containing cavity of their respective complexes to produce functional cleaved fragments (Kopan and Illagan, 2004).

The cytotoxic effect of GSI1 and MG132 on breast cancer cells is different (Figure 6C). As GSI1, MG132 induces G2/M arrest and apoptosis with loss of Bcl-2 expression (Yan *et al*, 2007), thus the therapeutic use of proteasome inhibitors are also being considered in cancer treatment (Voorhees *et al*, 2003; Sato *et al*, 2008). Whether the uptake of these compounds by breast cancer cells or their half-lives (either in the culture medium or intracellularly) is different, and thus modulate their potency, remains to be established.

Two facts are important from a potential future clinical application of GSI1: (1) the inhibitor is effective in triggering cell death of cancer cell lines at much lower concentrations than those required in non-tumorigenic cell lines, and (2) its effect on breast cancer cells is irrespective of their ER status. This last point is particularly important as triple-negative breast tumours (ER-, progesterone receptor-, and Her-2-negative), which are particularly aggressive and have a poor prognosis, are currently only treated with traditional chemotherapy (Haffty *et al*, 2006; Cleator *et al*, 2007). Interestingly, *γ*-secretase inhibitors have the potential of increasing sensitivity and efficacy of other chemotherapeutic drugs as well as hormonal and targeted therapeutic agents. For example, synergistic treatment of breast cancer cells with a *γ*-secretase inhibitor and trastuzamub (Osipo *et al*, 2008) or Tamoxifen (Rizzo *et al*, 2008) was more effective in reducing proliferation than the individual treatments. The combined treatments of *γ*-secretase inhibitors and chemotherapeutic agents have illustrated a greater extent of antiproliferative effects and/or apoptosis in multiple myeloma (Nefedova *et al*, 2008), T-cell acute lymphoblastic leukaemia (De Keersmaecker *et al*, 2008), and colon cancer cell lines (Akiyoshi *et al*, 2008).

Overall these data indicate that *γ*-secretase is a potential therapeutic target in breast cancer.
