# Supplementary figures and images for: Inhibition of γ-secretase induces G2/M arrest and triggers apoptosis in breast cancer cells
Source: Br J Cancer. 2009 Jun 9;100(12):1879–88. doi: 10.1038/sj.bjc.6605034 (PMC2714234; doi:10.1038/sj.bjc.6605034)

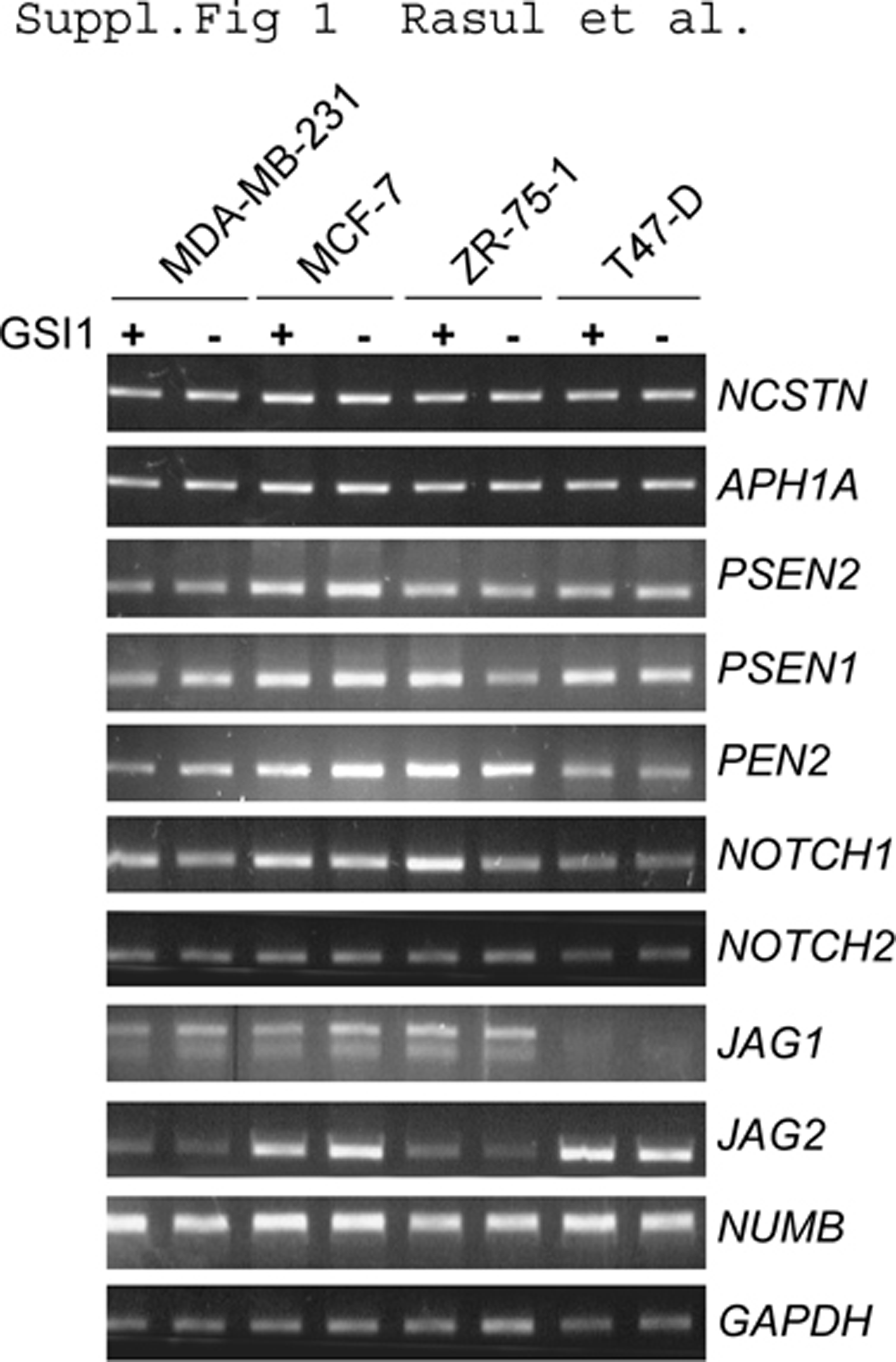

Supplement: Supplementary Figure 1 [file 6605034x1.tif]
