# Supplementary material for: Inhibition of γ-secretase induces G2/M arrest and triggers apoptosis in breast cancer cells
Source: Br J Cancer. 2009 Jun 9;100(12):1879–88. doi: 10.1038/sj.bjc.6605034 (PMC2714234; doi:10.1038/sj.bjc.6605034)
Supplement: Supplementary Figure Legend [file 6605034x2.doc]

**Supplementary Figure 1.** GSI1 treatment does not change the expression of genes coding for γ-secretase components or its targets. Cells were treated with 0.75 µM GSI1 for 48 h and the expression of the genes monitored by semi-quantitative RT-PCR.
