# Supplementary material for: Inhibition of γ-secretase induces G2/M arrest and triggers apoptosis in breast cancer cells
Source: Br J Cancer. 2009 Jun 9;100(12):1879–88. doi: 10.1038/sj.bjc.6605034 (PMC2714234; doi:10.1038/sj.bjc.6605034)
Supplement: Supplementary Table 1 [file 6605034x3.doc]

**Supplementary Table 1.**  Oligonucleotides and PCR conditions used.

| Gene | Forward primer  (5’ to 3’) | Reverse primer  (5’ to 3’) | MgCl_2_  (mM) | Denaturation^a^  °C /s | Annealing °C / s | Elongation  °C / s | Number of cycles | Product  Size (bp) |
| --- | --- | --- | --- | --- | --- | --- | --- | --- |
| *NOTCH1* | gcaacagctccttccacttc | ccacgaagaacagaagcaca | 3 | 95/25 | 59/27 | 72/25 | 34 | 499 |
| *NOTCH2* | atgactgccctaaccacagg | ctggagtacaggaggcgaag | 2 | 95/15 | 59/20 | 72/15 | 25 | 264 |
| *APH1A* | ccgctttgcctactacaagc | ccaaaaggtatggagcagga | 3 | 95/20 | 58/20 | 72/20 | 27 | 265 |
| *NCSTN* | caaagcaccttcagcatcaa | cgagctgccaatgtagtcaa | 3 | 95/25 | 58/25 | 72/25+86/2 | 28 | 315 |
| *PSEN1* | gttccacttcgtatgctggt | gcgaggatactgctggaaag | 3 | 95/20 | 60/15 | 72/20 | 23 | 314 |
| *PSEN2* | ctgcccaggagagaaatgag | cagtcaagggaggctcaaag | 3 | 95/25 | 61/25 | 72/25 | 25 | 198 |
| *PEN2* | tcccttgtcccagcctacac | ggtcctttattgggggatgt | 3 | 95/20 | 59/20 | 72/20 | 32 | 329 |
| *JAG1* | ctcctgtcgggatttgttta | ccacagacgttggaggaaat | 3 | 95/30 | 58/30 | 72/30 | 28 | 414 |
| *JAG2* | gtcaaggtggagacggttgt | atcctcgtcctcctcatcct | 2 | 95/30 | 60/30 | 72/30 | 25 | 345 |
| *NUMB* | agccagcccatactgctcta | acaggctgagaggtgaggaa | 2 | 95/30 | 60/30 | 72/30 | 30 | 346 |
| *GAPDH* | tctcatcaccatcttcca | catcacgccacagtttcc | 2 | 95/30 | 54/30 | 72/30 | 32 | 380 |

^a^An initial step (hot start) of 95°C for 10 min was carried out before amplification cycles.
