# Supplementary material for: Inhibition of γ-secretase induces G2/M arrest and triggers apoptosis in breast cancer cells
Source: Br J Cancer. 2009 Jun 9;100(12):1879–88. doi: 10.1038/sj.bjc.6605034 (PMC2714234; doi:10.1038/sj.bjc.6605034)
Supplement: Supplementary Table 2 [file 6605034x4.doc]

**Supplementary Table 2**. Summary of results from NCI60 Cell Screen

____________________________________________________________________________________________________________________

Run 1 Run 2

__________________________________ __________________________________

Cell Line Log_10_ GI50 Log_10_ TGI Log_10_ LC50 Log_10_ GI50 Log_10_ TGI Log_10_ LC50

(M) (M) (M) (M) (M) (M)

____________________________________________________________________________________________________________________

MCF7 -6.21 -4.41 >-4.00 -6.31 -4.95 -4.21

MDA-MB-231 -6.28 -5.64 -5.11 -6.43 -5.79 -4.02

HS 578T -6.35 -4.50 >-4.00 -6.68 -6.26 >-4.00

MDA-MB-435 -6.56 -6.15 -5.14 -6.79 -6.51 -6.22

BT-549 -6.29 -5.45 >-4.00 -6.48 -5.72 -5.01

T47-D -5.73 >-4.00 >-4.00 -6.60 -6.05 >-4.00

MDA-MB-468 -6.79 -6.45

Mean Breast Cancer Cell Lines -6.23±0.063 -5.23±0.12 -5.12±0.078 -6.58±0.059 -5.96±0.087 -4.86±0.12

Mean All Cell Lines -6.13±0.007 -5.02±0.014 -4.41±0.011 -6.41±0.005 -5.72±0.011 -4.82±0.014

____________________________________________________________________________________________________________________
